# Age, time from injury to surgery and hop performance after primary ACLR affect the risk of contralateral ACLR

**DOI:** 10.1007/s00167-021-06759-6

**Published:** 2021-10-07

**Authors:** Riccardo Cristiani, Magnus Forssblad, Gunnar Edman, Karl Eriksson, Anders Stålman

**Affiliations:** 1 Department of Molecular Medicine and Surgery, Stockholm Sports Trauma Research Center, Karolinska Institutet, Stockholm, Sweden; 2 Capio Artro Clinic, FIFA Medical Centre of Excellence, Sophiahemmet Hospital, Valhallavägen 91, 11486 Stockholm, Sweden; 3 Department of Orthopaedics, Stockholm South Hospital, Karolinska Institutet, Stockholm, Sweden

**Keywords:** Anterior cruciate ligament, ACL, Contralateral, Age, Graft, Meniscus, Cartilage, Quadriceps strength, Limb symmetry index

## Abstract

**Purpose:**

To evaluate factors affecting the risk of contralateral anterior cruciate ligament reconstruction (ACLR) within 5 years of primary ACLR.

**Methods:**

Primary ACLRs performed at Capio Artro Clinic, Stockholm, Sweden, during the period 2005–2014, were reviewed. The outcome of the study was the occurrence of contralateral ACLR within 5 years of primary ACLR. Univariable and multivariable logistic regression analyses were employed to identify preoperative [age, gender, body mass index (BMI), time from injury to surgery, pre-injury Tegner activity level], intraoperative [graft type, medial meniscus (MM) and lateral meniscus (LM) resection or repair, cartilage injury] and postoperative [limb symmetry index (LSI) for quadriceps and hamstring strength and single-leg-hop test performance at 6 months] risk factors for contralateral ACLR.

**Results:**

A total of 5393 patients who underwent primary ACLR were included. The incidence of contralateral ACLR within 5 years was 4.7%. Univariable analysis revealed that age ≥ 25 years, BMI ≥ 25 kg/m^2^, time from injury to surgery ≥ 12 months and the presence of a cartilage injury reduced the odds, whereas female gender, pre-injury Tegner activity level ≥ 6, quadriceps and hamstring strength and a single-leg-hop test LSI of ≥ 90% increased the odds of contralateral ACLR. Multivariable analysis showed that the risk of contralateral ACLR was significantly affected only from age ≥ 25 years (OR 0.40; 95% CI 0.28–0.58; *P* < 0.001), time from injury to surgery ≥ 12 months (OR 0.48; 95% CI 0.30–0.75; *P* = 0.001) and a single-leg-hop test LSI of ≥ 90% (OR 1.56; 95% CI 1.04–2.34; *P* = 0.03).

**Conclusion:**

Older age (≥ 25 years) and delayed primary ACLR (≥ 12 months) reduced the odds, whereas a symmetrical (LSI ≥ 90%) 6-month single-leg-hop test increased the odds of contralateral ACLR within 5 years of primary ACLR. Knowledge of the factors affecting the risk of contralateral ACLR is important when it comes to the appropriate counselling for primary ACLR. Patients should be advised regarding factors affecting the risk of contralateral ACLR.

**Level of evidence:**

Level III.

## Introduction

An anterior cruciate ligament (ACL) tear is a major knee injury which might have a serious impact on patients’ quality of life. An ACL reconstruction (ACLR) is generally recommended for patients involved in cutting/pivoting activities or in the event of functional instability [28]. Primary ACLR is successful in restoring knee laxity and improving subjective knee function [7, 8, 11, 15]. However, patients with an ACL-reconstructed knee run a greater risk of sustaining an ACL injury in either knee compared with individuals with a healthy knee [26]. In particular, the contralateral knee runs a higher risk compared with the ipsilateral knee at longer follow-ups [39]. Previous studies have reported rates of contralateral ACL injury ranging from 2 to 15% [2, 3, 20, 39], with this event typically occurring between 1 and 4 years after the primary ACLR [37, 39]. Several patients suffering a contralateral ACL injury may undergo ACLR. Data from the Swedish National Knee Ligament Registry [19] showed a contralateral ACLR rate of 3.8% at a 5-year follow-up after primary ACLR. Even though a recent study [12] reported that the results in terms of knee laxity and subjective knee function after contralateral ACLR are comparable to those after primary ACLR, contralateral ACLR remains a troublesome event for patients, as it requires the repeat of another long rehabilitation process.

A knowledge of the risk factors for contralateral ACLR is therefore important for clinicians when counselling patients undergoing primary ACLR. In addition, if some of these factors are modifiable, there would be the possibility to reduce the risk of this serious event.

There is evidence from several studies [2, 16, 17, 23, 31, 36] that younger age is an important risk factor for contralateral ACLR. However, the literature is scarce and inconsistent regarding the role of gender [2, 16, 23, 31, 36], body mass index (BMI) [13, 23], time from injury to primary ACLR [2, 13] and the graft choice at primary ACLR [2, 23, 27]. Moreover, there is a lack of studies investigating the potential influence on the risk of contralateral ACLR of factors that are modifiable by rehabilitation, such as muscle strength and hop performance after primary ACLR.

The purpose of this study was to evaluate risk factors for contralateral ACLR within 5 years of primary ACLR. It was hypothesised that female gender, younger age, a high (≥ 6) pre-injury Tegner activity level [33], the use of a bone-patellar tendon-bone (BPTB) autograft at the time of primary ACLR and a symmetrical (limb symmetry index [LSI] of ≥ 90%) quadriceps strength and hop test performance 6 months after primary ACLR would be risk factors for contralateral ACLR within 5 years after the index surgery.

## Materials and methods

Patient data were extracted from our clinic database. Patients registered for primary ACLR from 2 January 2005 to 7 March 2014 were assessed for eligibility. Only patients who underwent primary ACLR with a hamstring tendon (HT) or a BPTB autograft and had no concomitant ligament injuries were included.

Ethical approval for this study was obtained from the Regional Ethics Committee, Karolinska Institutet (Dnr 2016/1613-31/2).

### Surgical technique and rehabilitation

A single-bundle technique was used for all primary ACLRs. Only patients who received an HT or a BPTB graft were included. The choice of the graft was made by the operating surgeon. Graft harvesting and fixation, femoral tunnel drilling as well as meniscal repair techniques have been described earlier [6, 8, 9]. Rehabilitation milestones have also been reported previously [6–9]. Patients were recommended to return to sport at the earliest 6 months postoperatively, depending on quadriceps and hamstring strength (LSI ≥ 90%) and the results of the single-leg-hop test (LSI ≥ 90%).

### Isokinetic strength and single-leg-hop test performance assessment

The assessment of isokinetic strength and single-leg-hop test performance was made 6 months postoperatively. A standardized protocol was used. The Biodex System 3 (Biodex Medical Systems, Shirley, New York, USA) was used for testing. Isokinetic strength (quadriceps and hamstring) was tested in both knees at 90°/sec and in range of motion of 90 °–10 ° of knee flexion. The test started always with the uninjured knee. Before the test, the patients warmed up using a stationary cycling ergometer at a low resistance for 10 min. A verbal description of the test was given and patients were allowed to perform 2–3 practical trials before the test. A total of 5 maximum contractions (quadriceps and hamstring) with each leg were performed by the patients. The highest torque values for both quadriceps and hamstring were registered.

Hop performance was assessed with the single-leg-hop test [6, 10, 35]. Patients stood on one leg and were asked to jump straight as far as possible and land on the same leg. A stable landing was the criterion to consider the test successful. The test was repeated in case of early touchdown with the contralateral leg, loss of balance or in the event that the patient took additional hops after landing. A verbal description of the test was provided to the patients. Some practical trials were also allowed, until patients felt enough confident with the modality of the test. Three trials were performed for each leg. The uninjured leg was always tested first. The trial in which was obtained the best result for each leg was registered.

### Data sources

Data were extracted from our clinic database. The *preoperative factors* evaluated were age, gender, body mass index (BMI), time from injury to primary ACLR and pre-injury Tegner activity level [33]. Age was dichotomised into unbiased classes close to the median (< 25 years or ≥ 25 years). The BMI was dichotomised at 25 kg/m^2^, since a BMI of ≥ 25 is considered overweight [38]. Time from injury to primary ACLR was dichotomised into unbiased classes (< 12 months or ≥ 12 months) close to the median. Finally, the pre-injury Tegner activity level was dichotomised as high (≥ 6) or low (< 6). The *intraoperative factors* investigated were the type of graft (HT or BPTB autograft), medial meniscus (MM) resection, lateral meniscus (LM) resection, MM repair, LM repair and cartilage injury. The *postoperative factors* (6 months) included were isokinetic quadriceps and hamstring strength tests and the single-leg-hop test. The results of these tests were reported depending on the limb symmetry index (LSI) as symmetrical (LSI of ≥ 90%) or asymmetrical (LSI of < 90%) [8, 9, 35].

### Outcome

The outcome was the occurrence of contralateral ACLR within 5 years of primary ACLR. Patients who underwent contralateral ACLR at any institution in the country were identified through their unique Swedish personal identity number [22] in the Swedish National Knee Ligament Registry [34]. Follow-up started on the date of the index, primary ACLR and was up to 5 years (1827 days). Patients who underwent contralateral ACLR within this lapse of time were identified and included in the analysis.

### Statistical analysis

Statistical analysis was performed using the Statistical Package for Social Sciences, SPSS (Version 25.0, IBM Corp., Armonk, New York, USA). Univariable analyses were performed with age (< 25 years vs. ≥ 25 years), gender, BMI (< 25 kg/m^2^ vs. ≥ 25 kg/m^2^), time from injury to primary ACLR (< 12 months vs. ≥ 12 months), pre-injury Tegner activity level (high ≥ 6 vs. low < 6), graft type (HT vs. BPTB autograft), MM resection, MM repair, LM resection, LM repair, cartilage injury, 6-month quadriceps and hamstring strength and single-leg-hop test performance (LSI ≥ 90% vs. LSI < 90%) as independent variables and contralateral ACLR as dependent variable. A multivariable logistic regression analysis was performed to evaluate independent risk factors for contralateral ACLR. Only variables attaining a *P* value of < 0.2 in the univariable analysis were entered in the multivariable analysis. The BMI was excluded from the multivariable model due to missing data. The results were reported as odds ratios (OR) with 95% confidence intervals (CI). The level of significance in all analyses was 5% (two tailed).

## Results

A total of 5393 patients who underwent primary ACLR were identified. A total of 252 patients (4.7%) underwent contralateral ACLR during the 5-year follow-up. Patient characteristics for both the no-contralateral ACLR (*n* = 5141) and contralateral ACLR (*n* = 252) groups are summarised in Table 1.

**Table 1:** Patient characteristics and factors associated with the risk of contralateral ACLR in univariable analysis

	No contralateral ACLR (*n* = 5141)	Contralateral ACLR (*n* = 252)	OR (95% CI)	*P* value
Preoperative factors				
Age at surgery, years, mean ± SD	28.7 ± 10.5	22.7 ± 9.3		
Age ≥ 25 years	2864 (55.7)	70 (27.8)	0.30 (0.23–0.41)	< 0.001*
Age < 25 years	2277 (44.3)	182 (72.2)		
Gender				
Female	2249 (43.7)	132 (52.4)	1.41 (1.10–1.82)	0.007*
Male	2892 (56.3)	120 (47.6)		
BMI, kg/m^2^, mean ± SD	24.2 ± 3.5	23.7 ± 3.1		
≥ 25	1108 (29.2)	47 (21.9)	0.68 (0.49–0.95)	0.02*
< 25	2693 (70.8)	168 (78.1)		
	*n* = 3801	*n* = 215		
Time from injury to surgery, months, mean ± SD	17.7 ± 31.1	8.2 ± 13.9		
≥ 12 months	1501 (31.1)	34 (14.0)	0.36 (0.25–0.52)	< 0.001*
< 12 months	3318 (68.9)	209 (86.0)		
	*n* = 4819	*n* = 243		
Pre-injury Tegner activity level, median (range)	7 (1–10)	8 (2–10)		
High, ≥ 6	4031 (88.2)	217 (95.6)	2.90 (1.53–5.49)	0.001*
Low, < 6	538 (11.8)	10 (4.4)		
	*n* = 4569	*n* = 227		
Intraoperative factors				
Graft type				
HT autograft	4719 (91.8)	239 (94.8)	1.18 (0.98–1.43)	0.08
BPTB autograft	422 (8.2)	13 (5.2)		
Medial meniscus surgery				
Resection	779 (15.2)	33 (13.1)	0.84 (0.58–1.23)	0.37
Repair	284 (5.5)	20 (7.9)	1.47 (0.92–2.36)	0.10
Lateral meniscus surgery				
Resection	815 (15.9)	42 (16.7)	1.06 (0.76–1.49)	0.73
Repair	179 (3.5)	12 (4.8)	1.39 (0.76–2.52)	0.28
Cartilage injury				
Yes	1026 (20.0)	37 (14.7)	0.69 (0.48–0.99)	0.04*
No	4115 (80.0)	215 (85.3)		
Postoperative factors (6 months)				
Isokinetic quadriceps strength LSI, mean ± SD	84.6 ± 16.2	88.5 ± 14.7		
≥ 90%	1495 (33.8)	95 (42.4)	1.44 (1.10–1.90)	0.008*
< 90%	2931 (66.2)	129 (57.6)		
	*n* = 4426	*n* = 224		
Isokinetic hamstring strength LSI, mean ± SD	90.0 ± 19.7	91.8 ± 15.7		
≥ 90%	2001 (45.3)	118 (52.7)	1.35 (1.03–1.76)	0.03*
< 90%	2419 (54.7)	106 (47.3)		
	*n* = 4420	*n* = 224		
Single-leg-hop test LSI, mean ± SD	92.5 ± 13.2	96.1 ± 9.5		
≥ 90%	2624 (67.1)	164 (80.0)	1.96 (1.38–2.77)	< 0.001*
< 90%	1284 (32.9)	41 (20.0)		
	*n* = 3908	*n* = 205		

Data are reported as *n* (%), unless otherwise indicated

*ACLR* anterior cruciate ligament reconstruction, *BMI* body mass index, *BPTB* bone-patellar tendon-bone, *CI* confidence intervals, *HT* hamstring tendon, *LSI* limb symmetry index, *OR* odds ratio, *SD* standard deviation

*Statistically significant (*P* < 0.05)

### Univariable analyses

Univariable logistic regression analyses revealed that older age (≥ 25 years) (OR 0.30; 95% CI 0.23–0.41; *P* < 0.001), BMI ≥ 25 kg/m^2^ (OR 0.68; 95% CI 0.49–0.95; *P* = 0.02), time from injury to primary ACLR > 12 months (OR 0.36; 95% CI 0.25–0.52; *P* < 0.001) and cartilage injury (OR 0.69; 95% CI 0.48–0.99; *P* = 0.04) decreased the odds of contralateral ACLR, whereas female gender (OR 1.41; 95% CI 1.10–1.82; *P* = 0.007), pre-injury Tegner activity level ≥ 6 (OR 2.90; 95% CI 1.53–5.49; *P* = 0.001), quadriceps strength LSI of ≥ 90% (OR 1.44; 95% CI 1.10–1.90; *P* = 0.008), hamstring strength LSI of ≥ 90% (OR 1.35; 95% CI 1.03–1.76; *P* = 0.03) and single-leg-hop test LSI of ≥ 90% (OR 1.96; 95% CI 1.38–2.77; *P* < 0.001) increased the odds. No significant relationship was found between contralateral ACLR and graft type, MM and LM resection or repair (Table 1).

### Multivariable analysis

Multivariable logistic regression analysis (included patients: 3,266 no-contralateral ACLR, 175 contralateral ACLR) revealed that older age (≥ 25 years) (OR 0.40; 95% CI 0.28–0.58; *P* < 0.001) and time from injury to primary ACLR of ≥ 12 months (OR 0.48; 95% CI 0.30–0.75; *P* = 0.001) reduced the odds, whereas a 6-month single-leg-hop test LSI of ≥ 90% (OR 1.56; 95% CI 1.04–2.34; *P* = 0.03) increased the odds of contralateral ACLR. No significant relationships were found between contralateral ACLR and female gender, pre-injury Tegner activity level of ≥ 6, HT autograft, MM repair, the presence of a cartilage injury and 6-month isokinetic quadriceps or hamstring strength LSI of ≥ 90% (Table 2).

**Table 2:** Factors associated with the risk of contralateral ACLR in multivariable logistic regression analysis

	Regression coefficient (*β*)	SE	OR (95% CI)	*P* value
Preoperative factors				
Age ≥ 25 years	− 0.90	0.19	0.40 (0.28–0.58)	< 0.001*
Female gender	0.19	0.16	1.21 (0.89–1.67)	0.21
Time from injury to surgery ≥ 12 months	− 0.73	0.23	0.47 (0.30–0.75)	0.001*
Pre-injury Tegner activity level ≥ 6	0.60	0.39	1.83 (0.84–3.99)	0.12
Intraoperative factors				
HT autograft	0.19	0.36	1.21 (0.59–2.45)	0.59
MM repair	0.16	0.30	1.17 (0.64–2.13)	0.59
Cartilage injury	− 0.07	0.24	0.92 (0.57–1.49)	0.92
Postoperative factors (6 months)				
Isokinetic quadriceps strength LSI ≥ 90%	0.04	0.16	1.04 (0.75–1.45)	0.80
Isokinetic hamstring strength LSI ≥ 90%	0.08	0.16	1.08 (0.79–1.49)	0.61
Single-leg-hop test LSI ≥ 90%	0.44	0.21	1.56 (1.04–2.34)	0.03*

*ACLR* anterior cruciate ligament reconstruction, *CI* confidence interval, *HT* hamstring tendon, *LSI* limb symmetry index, *MM* medial meniscus, *OR* odds ratio, *SE* standard error

*Statistically significant (*P* < 0.05)

## Discussion

The most important finding in this study was that older age (≥ 25 years) and delayed primary ACLR (≥ 12 months) reduced the odds, whereas a symmetrical (LSI ≥ 90%) 6-month single-leg-hop test increased the odds of contralateral ACLR within 5 years of primary ACLR. In the entire cohort, the incidence of contralateral ACLR was 4.7% within 5 years of the index surgery.

In the literature, there is consistent evidence that younger age is an important risk factor for contralateral ACLR [2, 16, 17, 23, 31, 36]. Younger patients are more likely to return to high-risk activities, involving cutting/pivoting movements after an ACLR [5, 23, 30, 37], which could make them more prone to contralateral ACL injuries compared with their older counterparts [4, 18, 20]. Moreover, younger patients may tolerate less knee instability after a contralateral ACL injury and might be more willing to undergo a contralateral ACLR to continue participating in their pre-injury activities [23, 30]. In line with previous literature, a reduced odd of contralateral ACLR was found in older (age ≥ 25 years) patients.

In the present study, female gender did not affect the risk of contralateral ACLR in the multivariable regression model, meaning that gender is probably not associated per se with contralateral ACLR. These results are supported by those of the large cohort registry studies by Andernord et al. [2], Wasserstein et al. [36] and Gallo et al. [16], which found that patient gender was not a predictor of contralateral ACLR. In contrast, the large cohort registry studies by Maletis et al. [23] and Snaebjornsson et al. [31] reported female gender as a factor associated with an increased risk of contralateral ACLR. Taken together, these results lead us to conclude that, to date, there is no definitive association between female gender and the risk of contralateral ACLR.

One poorly investigated risk factor for contralateral ACLR is the delay of primary ACLR. Andernord et al. [2] reported that, in female patients, an index ACLR performed within 6 months of the injury was a predictor of contralateral ACLR. Similarly, Fältström et al. [14] found that an early ACLR (< 3 months from injury to surgery) was a predictor of contralateral ACLR. However, although the results of both studies were adjusted for age, they did not take account of the patient’s preinjury activity level. The authors [2, 14] suggested that, since an early ACLR in Sweden is predominantly performed on individuals with high preinjury activity levels, the injury to surgery interval was probably a proxy measurement of the patient’s activity level.

In the current study, a pre-injury Tegner activity level ≥ 6 increased the odds of having a contralateral ACLR in the univariable analysis. However, the multivariable regression model did not show any relationship between pre-injury activity level and contralateral ACLR. Conversely, delayed primary ACLR (≥ 12 months) decreased the risk of having contralateral ACLR. It has previously been hypothesised [1, 6, 32] that patients with delayed primary ACLR may adapt to the injured knee, thereby having less risk to expose their operated knee to re-injuries and revision ACLR. The same consideration could be applied for the risk of contralateral ACL injury and reconstruction. Moreover, as reported for revision ACLR [1, 6], another hypothesis may be that patients undergoing early primary ACLR would be more willing to undergo also early contralateral ACLR to continue performing their pre-injury activities. This would then bias the results indicating lower risk of contralateral ACLR in case of delayed primary ACLR.

In this study, no correlation was found between cartilage injury and repair or resection of the MM or LM at the time of the primary ACLR and the occurrence of contralateral ACLR. These results are in line with those of previous studies showing that neither meniscus nor cartilage injuries [2, 29] or meniscus procedures [4, 36] performed at the primary ACLR affect the risk of contralateral ACL injury or reconstruction.

There is still controversy about the effect of graft choice at primary ACLR as a possible risk factor for contralateral ACL injury or reconstruction. Conflicting results have been reported in both RCTs and registry studies. In an RCT based on 330 patients with a minimum 2-year follow-up, Mohtadi et al. [24] found no differences in the rate of contralateral ACL injury between patients with HT or BPTB autografts. On the other hand, in an RCT based on 90 patients with a 15-year follow-up, Leys et al. [20] reported that the use of a BPTB autograft rather than an HT autograft was associated with 2.6 times higher odds of contralateral ACL injury. In a large cohort study based on the Swedish national knee ligament registry, Andernord et al. [2] showed that the choice between an HT or a BPTB autograft at the primary ACLR did not affect the risk of contralateral ACLR at a 5-year follow-up. In contrast, in a recent study based on the New Zealand ACL registry, Rahardja et al. [27] reported that patients with a BPTB autograft at the primary ACLR ran a 1.9 times higher risk of undergoing a contralateral ACLR compared with patients with an HT autograft. The authors included several variables in their Cox proportional hazard regression analysis. Adjustments were made for age, gender, time from injury to primary ACLR, activity at the time of injury, meniscus treatment and preoperative Marx activity score. In the present study, neither the univariable nor the multivariable logistic regression analysis (including age, gender, time from injury to primary ACLR, pre-injury Tegner activity level, MM repair, cartilage injury, 6-month isokinetic quadriceps and hamstring strength and single-leg-hop test performance) revealed any significant association between graft choice and the risk of undergoing contralateral ACLR, suggesting that the graft at primary ACLR is probably not associated per se with the incidence of contralateral ACLR. To date, based on these conflicting results, it is difficult to draw any definitive conclusion regarding the relationship between graft choice at primary ACLR and the risk of contralateral ACLR.

The significant association between symmetrical (LSI of ≥ 90%) isokinetic quadriceps or hamstring strength 6 months after the index surgery (primary ACLR) and contralateral ACLR in the univariable analysis was not present in the multivariable analysis. A recent large cohort study [9] showed that older age reduced the odds of achieving symmetrical isokinetic quadriceps and hamstring strength 6 months after primary ACLR. Older age (≥ 25 years) resulted to be the factor that most affected the risk of contralateral ACLR in the multivariable analysis. This may suggest that the results of these tests probably do not have an important effect on the risk of contralateral ACLR, whereas age at the time of primary ACLR is a significant risk factor.

One interesting result was that a symmetrical (LSI ≥ 90%) single-leg-hop test performance 6 months after the index surgery (primary ACLR) was a factor that independently affected the risk of contralateral ACLR. Single-leg-hop tests are commonly used to assess confidence in the limb, neuromuscular control and the ability to tolerate loads related to sport activities [21]. It has been shown that hop tests 6 months after ACLR are able to predict return to sport participation at the preinjury activity level [25]. It might be hypothesised that patients who achieved a single-leg-hop test LSI of ≥ 90% may have returned to their preinjury activity level to a greater extent compared with patients with an LSI of < 90%. This may have exposed their contralateral knee to a higher risk of ACL injury and subsequent ACLR.

The major strength of the current study was the large cohort included (5393 patients). Moreover, there was a large (*n* = 252) number of patients with contralateral ACLR. This allowed a comprehensive evaluation of several (preoperative, intraoperative and postoperative) risk factors for contralateral ACLR and gave the opportunity to conduct a robust multivariable logistic regression analysis. The latter is a crucial aspect when it comes to simultaneously and independently evaluating several potential risk factors for the outcome, as there might be an overlap between the factors investigated in the univariable analysis and it is essential to determine the relative importance of one factor over another. The large number and heterogeneity of the included patients give the results high external validity. Finally, another strength was that, in contrast to previous large cohort studies based on national registries, patients underwent ACLR and were assessed at the same clinic 6 months postoperatively. In addition, rehabilitation and recommendations for return to sport or preinjury activities were standardised.

There are some limitations to consider. The outcome of this study was contralateral ACLR and not contralateral ACL injury. Probably, not all patients with a contralateral ACL injury undergo ACLR. The number of contralateral ACLRs is therefore probably lower than that of contralateral ACL injuries and the risk factors for contralateral ACLR might differ from those for contralateral ACL injury. However, as reported by Andernord et al. [2], it is also important to consider that contralateral ACLR might be a more accurate measurement of the clinically disabling condition compared with contralateral ACL injury, as it might represent the proportion of patients who are not able to cope with their ACL-deficient knee. Missing data for some of our risk factors was also a limitation. The BMI (which was associated with a contralateral ACLR in the univariable analysis) had the highest proportion of missing values, which prevented its inclusion in the multivariable logistic regression analysis. These factors may have had an effect on the results. However, this limitation is probably reduced by the large cohort studied and the inclusion of many, different potential risk factors for contralateral ACLR. Finally, data on other factors which may potentially influence the risk of contralateral ACLR, such as activity level after the index surgery (primary ACLR) and return to sport, were not available and could therefore not be included in the analysis.

## Conclusion

Older age (≥ 25 years) and delayed primary ACLR (≥ 12 months) reduced the odds, whereas a symmetrical (LSI ≥ 90%) 6-month single-leg-hop test increased the odds of contralateral ACLR within 5 years of primary ACLR. Knowledge of the factors affecting the risk of contralateral ACLR is important when it comes to the appropriate counselling for primary ACLR. Patients should be advised regarding factors affecting the risk of contralateral ACLR.
